# Non-invasive and accurate risk evaluation of cerebrovascular disease using retinal fundus photo based on deep learning

**DOI:** 10.3389/fneur.2023.1257388

**Published:** 2023-09-07

**Authors:** Lin An, Jia Qin, Weili Jiang, Penghao Luo, Xiaoyan Luo, Yuzheng Lai, Mei Jin

**Affiliations:** ^1^Guangdong Weiren Meditech Co., Ltd, Foshan, Guangdong, China; ^2^Foshan Weizhi Meditech Co., Ltd, Foshan, Guangdong, China; ^3^Department of Ophthalmology, Guangdong Provincial Hospital of Integrated Traditional Chinese and Western Medicine, Foshan, Guangdong, China; ^4^Department of Neurology, Guangdong Provincial Hospital of Integrated Traditional Chinese and Western Medicine, Foshan, Guangdong, China

**Keywords:** deep learning, cerebrovascular disease, stroke, artificial intelligence, attention

## Abstract

**Background:**

Cerebrovascular disease (CeVD) is a prominent contributor to global mortality and profound disability. Extensive research has unveiled a connection between CeVD and retinal microvascular abnormalities. Nonetheless, manual analysis of fundus images remains a laborious and time-consuming task. Consequently, our objective is to develop a risk prediction model that utilizes retinal fundus photo to noninvasively and accurately assess cerebrovascular risks.

**Materials and methods:**

To leverage retinal fundus photo for CeVD risk evaluation, we proposed a novel model called Efficient Attention which combines the convolutional neural network with attention mechanism. This combination aims to reinforce the salient features present in fundus photos, consequently improving the accuracy and effectiveness of cerebrovascular risk assessment.

**Result:**

Our proposed model demonstrates notable advancements compared to the conventional ResNet and Efficient-Net architectures. The accuracy (ACC) of our model is 0.834 ± 0.03, surpassing Efficient-Net by a margin of 3.6%. Additionally, our model exhibits an improved area under the receiver operating characteristic curve (AUC) of 0.904 ± 0.02, surpassing other methods by a margin of 2.2%.

**Conclusion:**

This paper provides compelling evidence that Efficient-Attention methods can serve as effective and accurate tool for cerebrovascular risk. The results of the study strongly support the notion that retinal fundus photo holds great potential as a reliable predictor of CeVD, which offers a noninvasive, convenient and low-cost solution for large scale screening of CeVD.

## Introduction

Cerebrovascular disease, which impacts the blood vessels supplying the brain, is a significant global cause of mortality and profound disability (1, 2). Considerable research has been conducted for early detection of cerebrovascular disease and the majority of them concentrates on using neuroimaging techniques to detect morphological alterations in brain (3). However, to enhance treatment approaches, a crucial area of research focus is identifying potential biomarkers within other organs. These biomarkers have the potential to reflect underlying small vessel changes occurring beyond the brain, which significantly contribute to the development and progression of cerebrovascular disease. Such endeavors promise to optimize treatment regimens and improve patient outcomes (4). In recent years, there have been many deep learning methods to predict strokes (5–7).

An increasing body of research indicates that the retina is a valuable indicator of cerebrovascular risk (8). This association can be attributed to the shared neurodevelopmental origin of the retina and brain (9). Despite the complexity of the cerebrovascular system’s structure, there is a prevailing belief that retinal vascular disease reflects pathological changes *in vivo* within the cerebrovascular system (10). Extensive investigations have assessed the correlation between retinal parameters and CeVD (10–13). Within this context, three primary categories of retinopathy have been linked to CeVD: hypertensive retinopathy, clinical retinal disease, and retinal microvascular abnormalities encompassing arteriovenous scratches, focal arteriolar stenosis, and a reduced ratio of arterioles diameter to venules diameter (11). Furthermore, retinal microvascular abnormalities have been associated with an increased risk of stroke and elevated stroke-related mortality. Longitudinal studies provide evidence that retinal vascular changes can predict the risk of patients developing clinical manifestations of cerebrovascular disease (14, 15). These findings underscore the potential of retinal assessments as a non-invasive means of predicting and monitoring cerebrovascular disease.

In recent studies, the potential of fundus imaging in predicting the risk of cerebrovascular disease (CeVD) has been highlighted, indicating promising applications (16). Utilizing non-mydriatic fundus photography enables the acquisition of high-quality retinal fundus images for pathological analysis, thereby facilitating the identification of patients at risk of developing CeVD. However, manual examination of fundus photos is time-consuming, prompting the need for automated analysis using deep learning algorithms, which offer practicality for real-world implementation (17, 18). The primary objective of this study was to develop an Efficient-Attention Network-based model for automatic CeVD risk prediction using Reina Fundus Photo images. The performance of this deep learning approach was extensively evaluated to assess its effectiveness as an aid in CeVD risk assessment.

## Methods

### Data collection

We retrospectively examine our electronic medical record from 2019 to 2022. The inclusion criteria are patients who have received color fundus check and at least one type of angiography scan (MRA/CTA/DSA) of brain vessel. Patients with missing image data or poor image quality were excluded. We further collected patient information including age, gender and blood pressure. Patients were diagnosed as cerebrovascular disease if they were confirmed by angiography to have at least one of the following conditions: major cerebral artery narrowed by more than 50%, large vessel occlusion, presence of aneurysm, presence of arteriovenous malformation and small-vessel disease. Patients free of the above conditions were defined as control group.

We identified a total of 409 patients from our electronic medical record who meet the inclusion criteria and pass the exclusion criteria. 133 patients were diagnosed with cerebrovascular disease and the remained 276 patients free of cerebrovascular disease were used as control group. We compared the baseline characteristics of the two groups and calculated their corresponding *p*-value. The baseline characteristics of the two groups are shown below in Table 1. It can be seen that age, gender, blood pressure level and hypertension state all showed a statistically significant difference between the two groups.

**Table 1:** Baseline characteristics of the disease and control groups.

	Disease	Control	*p*-value
Age	72.9 ± 10.1	45.1 ± 12.2	<0.001
Systolic pressure	138.1 ± 17.7	128.2 ± 18.4	<0.001
Diastolic pressure	84.2 ± 12.5	77.6 ± 10.2	<0.001
Hypertension	75	60	<0.001
Gender (male)	58	66	<0.001

### Model development

**Figure 1:**
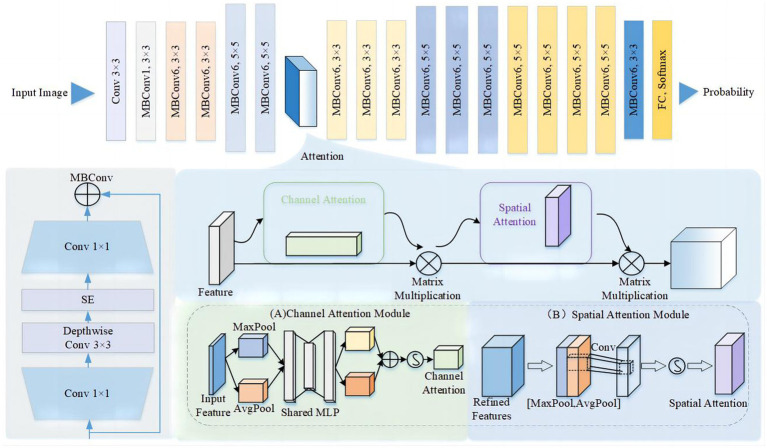
Network architecture of the convolutional block attention module.

**Figure 2:**
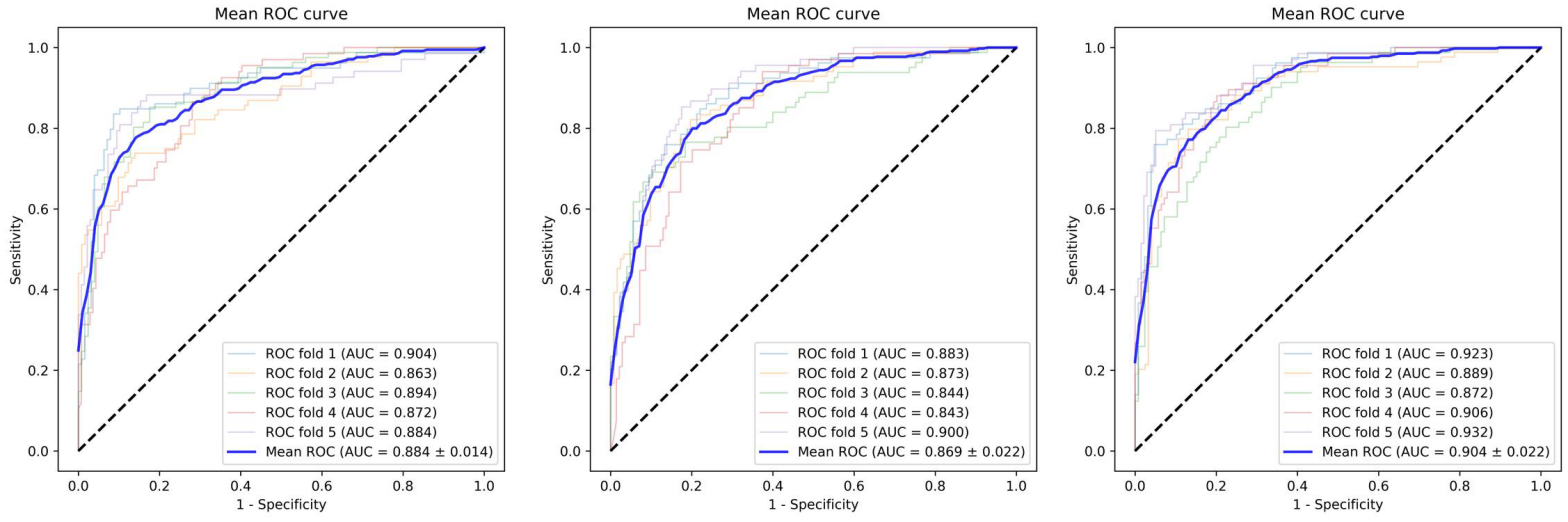
Averaged performance of the three models in 5-fold cross-validation.

In our study, we introduced an Efficient-Attention network based on Efficient-Net to enable accurate automatic prediction of CeVD risk using retinal fundus photos. EfficientNet (19) is renowned for its innovative scaling method, which balances network depth, width, and resolution to enhance model performance. However, effectively capturing spatial and channel attention is critical in cerebrovascular risk prediction (Figure 1). To address this, we incorporated the Convolutional Block Attention Module (CBAM) into our model architecture. The CBAM module facilitates affinity matrix learning on both channel and spatial dimensions, enabling the identification of features that contribute significantly to cerebrovascular risk prediction (20). The network architecture is shown in Figure 2. Furthermore, we visualized the extracted features to enhance the interpretability of the Efficient-Attention model, providing insights into the key factors driving the predictions.

The Efficient-Attention cerebrovascular risk prediction model was developed using the color image data from fundus photo. The training process of the model consists of two primary stages: data preprocessing and network learning. During the preprocessing stage, the pixel values of each retinal fundus photo are normalized to ensure they fall within the range of 0 to 1. In the stage of network learning, the images were resized to a resolution of 512 × 512, and the network was trained using the backpropagation (BP) algorithm (21). All networks were trained using ADAM optimizer in Pytorch 1.9.0, with an initial learning rate of 0.0003 for a total epoch of 100.

To address the limitations associated with small-sample independent datasets, we employed a five-fold cross-validation approach to evaluate the performance of our model. In cross-validation, all data is divided into five subsets and five rounds of testing are performed. In each round, four subsets were used as training data and the remaining one subset was used as test set. This process iterated until all subsets have been used as test set at least once. We obtained the final result by averaging the performance across these iterations, thereby mitigating the influence of specific dataset characteristics. Cross-validation enables a more comprehensive assessment of the model’s effectiveness. All models in this study were implemented in Python using the PyTorch deep learning framework (22).

## Results

### Model performance on CeVD risk prediction

During the CeVD risk prediction task, we employed several indicators to assess the accuracy of the Efficient-Attention model, including accuracy (ACC), the area under the curve (AUC), Specificity, and Sensitivity (23). These metrics were calculated to quantify the performance of our proposed model. To ensure a comprehensive evaluation, we compared the performance of our model with that of the classic EfficientNet and ResNet (24) models. To avoid over-optimistic result, we performed our evaluation on 5-fold cross-validation. All value reported below are the averaged performance during the 5 runs.

In our study, we developed a CeVD risk prediction model utilizing Reina Fundus Photo. The evaluation metrics used to quantify the risk assessment are presented in Table 2. Comparing our model with ResNet and EfficientNet, we achieved impressive results with ACC being 0.834 ± 0.03, AUC being 0.904 ± 0.02, and specificity being 0.884 ± 0.08. Compared to EfficientNet, our model exhibited 0.036, 0.021, and 0.70 improvements in ACC, AUC, and specificity, respectively. While our model may not have achieved the best sensitivity index, the Receiver Operating Characteristic (ROC) curve, as depicted in Figure 2, demonstrates that our model outperforms classical models in accurately predicting CeVD risk. The combined results from the ROC curve further validate the efficiency and effectiveness of our model in achieving precise CeVD risk prediction. These findings underscore the potential of our model to outperform classical approaches (ResNet and EfficinentNet).

**Table 2:** Performance comparison (ACC, AUC, specificity, and sensitivity) of different risk prediction models.

Model	ACC	AUC	Specificity	Sensitivity
ResNet	0.797 ± 0.02	0.868 ± 0.02	0.872 ± 0.06	0.678 ± 0.07
EfficientNet	0.798 ± 0.03	0.883 ± 0.01	0.814 ± 0.08	**0.772 ± 0.07**
Efficient-attention (our)	**0.834 ± 0.03**	**0.904 ± 0.02**	**0.884 ± 0.08**	0.746 ± 0.07
Efficient-attention (without spatial attention)	0.818 ± 0.03	0.893 ± 0.02	0.864 ± 0.05	0.733 ± 0.07
Efficient-attention (without channel attention)	0.821 ± 0.02	0.890 ± 0.02	0.878 ± 0.06	0.767 ± 0.07

The best value for each column/row is highlighted in bold. [Continuous variables were presented as mean ± standard deviation (SD) to provide a measure of the central tendency and dispersion].

### Model interpretation

**Figure 3:**
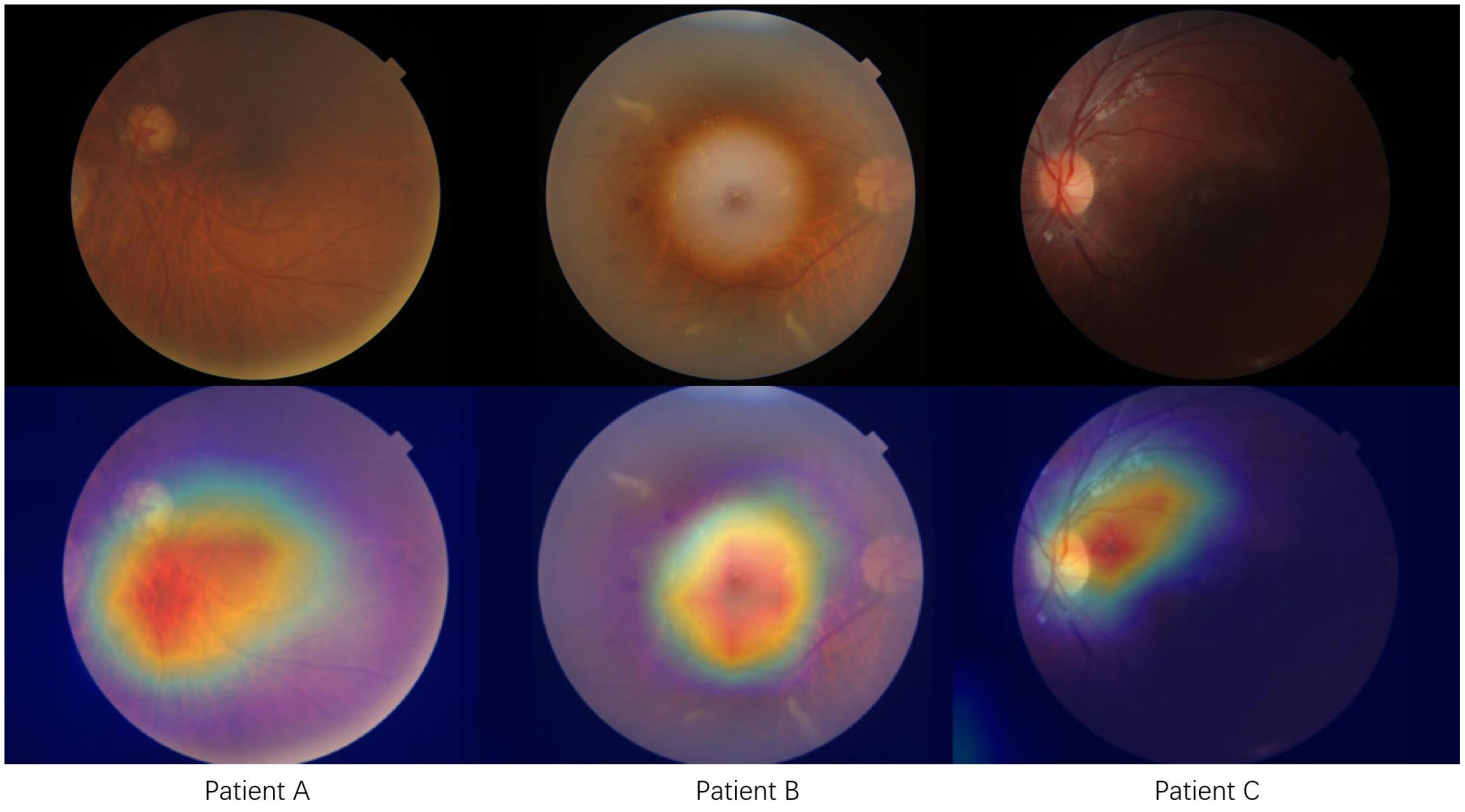
Model interpretation using Grad-CAM shows three typical cases with cerebrovascular disease. Red area indicates the activated or attention zone of our proposed model.

The interpretability of Efficient-Attention networks can be achieved through the application of Gradient-weighted Class Activation Mapping (Grad-CAM). Figure 3 in our study demonstrates the rationale behind our model by highlighting the most crucial features associated with predicting cardiovascular disease. We can observed that the model focuses extensively on retinal abnormalities presented on the fundus photo. This is consistent with finding from literature that retinal signs such as hemorrhage and exudates are correlated with increased stroke risk. By leveraging Grad-CAM, we gain insights into the specific regions within the fundus photo that contribute significantly to the prediction process. Implementing our Efficient-Attention network enhances feature learning and attention mechanisms specifically tailored for cerebrovascular risk prediction. This improved feature learning and attention allocation contribute to the enhanced performance of risk prediction models.

### Statistical analysis

All variables between the normal and diseased groups were compared using univariate analyses. For binary or categorical variables, Fisher exact test or chi-square test was performed. For continuous variables, they were first examined by the Shapiro–Wilk test to determine normality, followed by the Student *t* test (for normally distributed variables) or Mann–Whitney *U*-test (for non-normally distributed variables). *p* value < 0.05 was considered to be statistically significant. The above analyses were performed using SPSS (IBM Corporation, United State).

## Discussion

In this study, we successfully developed a CeVD risk prediction model based on retinal fundus photo using deep learning techniques. This model not only improves risk assessment performance but also enhances interpretability, making it a valuable tool in the field.

Traditionally, manual diagnosis and analysis methods for assessing retinal vascular abnormalities are tedious and time-consuming. However, deep learning has shown promising results in evaluating retinal images for cardiovascular risk prediction. Previous studies have demonstrated the association between retinal abnormalities and incident stroke, emphasizing the potential of retinal assessment as a target for CeVD prevention and treatment strategies (25, 26).

In our model, we built upon the well-known EfficientNet architecture by incorporating an Attention mechanism. This addition allows our model to capture features from both spatial and channel dimensions, enhancing its ability to predict CeVD risk accurately. Consequently, our model performed better than previous studies, with an ACC of 0.834 ± 0.03, AUC of 0.904 ± 0.02, and specificity of 0.884 ± 0.08. We employed Grad-CAM to visualize the model to enhance interpretability further, providing insights into the essential regions and features contributing to the prediction.

The outcomes derived from this research are anticipated to facilitate the utilization of deep learning techniques and fundus photography in the evaluation of cerebrovascular risk in the population of China. It is imperative to develop an appropriate risk screening methodology, which can facilitate early efficacious preventive interventions, such as the use of statins therapy (27). Furthermore, the application of the conventional stroke prediction model may become more challenging due to the restricted consultation duration, limited to a mere 2.0 min, in primary care physician settings. Conversely, developed countries exhibit an average consultation duration exceeding 20 min (28). In contrast, the use of non-mydriatic fundus photography, a non-invasive diagnostic tool operable by nonprofessional individuals, presents a viable alternative. Moreover, this examination requires less than 1 min and costs approximately 40 RMB yuan (roughly 6 US dollars) in China (29).

The ultimate goal of the current study is to facilitate the integration of this algorithm into primary healthcare settings, including community hospitals and health centers, wherein it can serve as an initial routine screening tool for identifying individuals at risk of CeVD. By implementing this algorithm, early detection and warning signals can be provided. Individuals identified as having borderline or higher risk through this algorithm should subsequently be advised to undergo further examinations targeting specific risk factors, such as blood pressure, blood sugar levels, and serum lipids.

Overall, our study highlights the promising application of deep learning in CeVD risk prediction and introduces a novel approach to CeVD prevention and treatment. By assessing and predicting CeVD risk through non-invasive and easily accessible fundus images, we offer a new pathway for early detection and intervention in CeVD cases.

### Limitation

There are several limitations in our current study. First, this is a retrospective study within a single center. Further validation using prospective cohort design with multicenter should be taken. Second, sample size is still relatively small. More cases should be included to strengthen the evidence. Third, only retinal images are used as input feature in the current study, a more comprehensive prediction model can be developed in the future to take other risk factors into account.

## Conclusion

Exploring retinal vascular abnormalities holds excellent potential for early detection of cerebrovascular events and could become a significant focus in CeVD prevention and treatment strategies. This article presents a CeVD risk prediction model based on Reina Fundus Photo, leveraging an Efficient-Attention architecture incorporating the Attention mechanism and Grad-CAM to enhance performance and interpretability. The proposed model outperforms classic models such as ResNet and EfficientNet regarding CeVD risk prediction. The model achieves higher performance by integrating the Attention mechanism and captures key features for accurate predictions. Additionally, Grad-CAM enables visualizations that provide insights into the model’s decision-making process, making it more interpretable.

This study demonstrates the promising potential of deep learning in CeVD risk prediction. By harnessing the power of deep learning and non-invasive imaging techniques, this method could pave the way for improved CeVD risk assessment and enable timely interventions to prevent and treat cerebrovascular events.

## Data availability statement

The dataset cannot be made public due to ethics requirement. Requests to access these datasets should be directed to MJ, jinmei75@163.com.

## Ethics statement

The studies involving humans were approved by Guangdong Provincial Hospital of Integrated Traditional Chinese and Western Medicine. The studies were conducted in accordance with the local legislation and institutional requirements. The Ethics Committee/Institutional Review Board waived the requirement of written informed consent for participation from the participants or the participants’ legal guardians/next of kin because this is a retrospective study and all patient cases have been anonymized and thus written consent was waived by IRB.

## Author contributions

LA: investigation, methodology, writing–review–and–editing, software. JQ: methodology, writing–review–and–editing, conceptualization. JW: writing – review and editing, methodology, writing – original draft. PL: methodology, software, writing – original draft. XL: data curation, formal analysis, writing – review and editing. YL: conceptualization, data curation, formal analysis, project administration, writing – review and editing. MJ: conceptualization, methodology, writing – review and editing, data curation, formal analysis, investigation, project administration.

## Funding

The author(s) declare financial support was received for the research, authorship, and/or publication of this article. This research is supported by the following funds: Foshan HKUST Projects (FSUST21-HKUST10E), Innovation and Entrepreneurship Teams Project of Guangdong Pearl River Talents Program (2019ZT08Y105), Guangdong Eye Intelligent Medical Imaging Equipment Engineering Technology Research Center (2022E076) and Medical Science and Technology Research Fund of Guangdong Province (A2022516).

## Conflict of interest

LA and JQ were employed by Guangdong Weiren Meditech Co., Ltd. WJ and PL was employed by Foshan Weizhi Meditech Co., Ltd.

The remaining authors declare that the research was conducted in the absence of any commercial or financial relationships that could be construed as a potential conflict of interest.

The handling editor CO declared a past co-authorship with the author WJ.

## Publisher’s note

All claims expressed in this article are solely those of the authors and do not necessarily represent those of their affiliated organizations, or those of the publisher, the editors and the reviewers. Any product that may be evaluated in this article, or claim that may be made by its manufacturer, is not guaranteed or endorsed by the publisher.
